# Supporting the Well-Being of People Living With Dementia and Their Family Carers Through Concurrent Arts and Well-Being Community Programs: Qualitative Perspectives of Participants and Facilitators

**DOI:** 10.1177/14713012251383967

**Published:** 2025-10-15

**Authors:** Carolyn M. Murray, Lenore de la Perrelle, Kerry Mart, John Baranoff, Geoff Richards, Gabrielle Rosa Hernandez, Angela Berndt

**Affiliations:** 1 Allied Health and Human Performance, University of South Australia, Adelaide, SA, Australia; 2 College of Nursing and Health Sciences, 1065 Flinders University, Bedford Park, SA, Australia; 3 Creative Beginnings Art, McLaren Vale, SA, Australia; 4 Faculty of Health and Medical Sciences, School of Psychology, University of Adelaide, Adelaide, SA, Australia; 5 Pathways Café, Mitchell Park, SA, Australia; 6 College of Health & Medicine, School of Health Sciences, University of Tasmania, Launceston, TAS, Australia

**Keywords:** dementia, family carers, art therapy, social isolation, carer well-being, respite, community connections, social support, qualitative, co-design

## Abstract

Community dwelling people with dementia and their family carers (dyads) may become increasingly isolated which can lead to lack of support, heightened stress levels, anxiety and difficulty coping. For both populations, there is a need for supportive and inviting programs that can provide social contact, respite, and promote engagement for well-being. A co-design workshop was conducted which led to the provision of a pilot six-week art program for people with dementia concurrent with a wellbeing program for their family carers. The research had two phases. Phase one was co-design informed by action research to decide on the content for the six-week concurrent programs and phase two used qualitative description to interpret participant perspectives about program outcomes. Data were collected *prior* to the programs through a co-design workshop, *during* the program through weekly reflections, and *after* program completion through interviews with dyads and a focus group with program facilitators. Twenty-one people participated in the co-design workshop which included two industry advocates, four caregivers, three people with dementia and twelve who did not specify. There were six dyads in the concurrent programs and six facilitators overall. Data were analysed using reflexive thematic analysis. Three themes developed in phase two included: relaxation, engagement and trust; recognising and developing new skills and abilities; and connecting over shared experiences. The co-designing process supported trust and led to programs that provided social support and opportunity for engagement. Having the dyads separated but nearby helped people to relax, knowing the other was doing something enriching. Concurrent programs for people with dementia and their caregivers must be tailored to their needs, have small group sizes that allow for social connection and trained facilitators that focus on the process of ‘doing’ activities and having fun over the outcome or product.

## Introduction

Dementia in the older population is increasing in prevalence with 9.9 million people developing dementia every year and by 2030 there will be approximately 75 million people worldwide living with dementia (World Health Organization, 2017). Because health is derived from a combination of physical, social, economic and contextual factors, the need to prevent social isolation and promote social support and well-being are becoming increasingly recognized (Chatterjee et al., 2018; Clements-Cortés & Yip, 2020; Hung et al., 2021). For people with memory loss and dementia, isolation may compound cognitive symptoms as well as lead to co-morbidities such as depression and, anxiety (Holt Lunstad, 2017; Sörensen & Conwell, 2011). People with dementia therefore need support to engage socially (Guideline Adaptation Committee, 2016), which is said to require a combination of involvement, inclusion, participation and engagement (Innes et al., 2024). Creative arts can play a major role in promoting health and well-being for people living with dementia through providing engagement and cognitive stimulation (Fancourt & Finn, 2019; Irons et al., 2020; Letrondo et al., 2023). For people living with dementia, arts activities can be personally meaningful, sustain attention, provide a sense of achievement and promote social interaction when implemented in groups (Cavalcanti Barroso et al., 2022).

Many people living in the community with dementia are being supported by unpaid caregivers who also have wellbeing and support needs. In 2021, there were approximately 700,000 unpaid caregivers of people living with dementia in the United Kingdom (Alzheimer’s Society, 2021) with numbers reaching over 11 million in the United States of America (U.S. Centers for Disease Control and Prevention, 2024) and over 140,000 in Australia (Australian Institute of Health and Welfare, 2024). Caregivers of people with dementia are providing essential support to enable the person being cared for to remain living at home. Most caregivers assume the role as a family responsibility and as care responsibilities increase with the progression of dementia, caregiver health and well-being are impacted (Australian Institute of Health and Welfare, 2024). The most unmet needs experienced by caregivers are access to sufficient respite, physical, social and emotional support, sleep and self-care (Australian Institute of Health and Welfare, 2024; Holt Clemmensen et al., 2021; Lauritzen et al., 2022; Waligora et al., 2018). Respite is defined as short term relief from caregiving to provide opportunity for taking a break and attending to personal well-being (National Institute on Aging (NIA), 2023).

Respite opportunities and support services are more likely to be accessed when they are flexible, tailored to individual needs, accept the heterogeneity of people with dementia, include open communication, allow control over the parameters of the respite and give people the time they need to build trust (Leocadie et al., 2018; Motta-Ochoa et al., 2021; Pinkert et al., 2021). Despite this much needed support for caregivers, there are access barriers including anxiety about separation from carers, worry about cost, and breakdowns in communication between service providers and recipients (Giebel et al., 2023). To fulfill this need, caregivers need to trust that the person with dementia will be safe and not stressed by the experience of being separated from them (Leocadie et al., 2018).

One means for flexible, attractive and restful respite and support opportunities that meet the needs of both the caregiver and the person with dementia is through provision of programs that involve them both. Certainly, Cavalcanti Barroso et al. (2022) found positive outcomes were achieved for people with dementia when arts activities were frequent, included both art and fun, used varied forms, and involved caregivers. The opportunity being created in this current research was an arts and well-being program to occur concurrently for both people living with dementia and their caregivers. The people with dementia participated in art and their caregivers engaged in wellbeing and social support activities. The concurrent program design kept the dyads close by, thus building trust and minimizing separation anxiety. Given the need to build trust and therefore engagement, the programs were developed using a co-design approach (Wang et al., 2019; Watt et al., 2024). Caregivers in this research are referred to as ‘family carers’ with ‘caregiver’ being used when discussing carers more broadly. When referring to both people with dementia and their family carers, the term ‘dyad’ will be used.

### Research Aim

As the research included two phases, there were two aims. The aim for phase one was to co-design concurrent well-being and art programs that promoted social support and provided a creative outlet for the participants. The second phase aimed to qualitatively explore perspectives of both participants and facilitators about their engagement and participation in the concurrent programs.

## Methods

### Research Design

*Phase one* of the research used a co-design approach that was informed by action research (Ayton, 2023; Lord et al., 2021; Robert et al., 2021) and *phase two* used a qualitative descriptive study to interpret perspectives of participants (Nayar & Stanley, 2023). The first phase involved a co-design workshop to develop the initial program content and inform planning and refinement of the program. Once dyads had expressed interest in the program, they participated in a ‘sign up’ day which was followed by the second phase of the project which was collection of program feedback through weekly reflections and a focus group with facilitators and interviews with dyads. In accordance with Australian Government requirements (Australian Research Council & National Health and Medical Research Council, 2025), ethical approval was provided by the University of South Australia (protocol number 205605) with reciprocal approval provided by Flinders University of South Australia and University of Adelaide. The COREQ criteria for quality reporting of qualitative studies was used (Tong et al., 2007).

### Context and Program Delivery

This research includes delivery of concurrently timed programs whereby people living with dementia participated in a six-week art program whilst their family carer participated in a well-being program. The concurrent programs took place in a community center in Trott Park, South Australia. As this was in the outer metropolitan area, there were some people who indicated they could not attend due to access issues. The art program was delivered by a skilled artist with dementia experience and the well-being program by skilled wellness coaches. There was no cost for attendance and the local council provided the venue without charge. The programs were co-designed with people with dementia, caregivers, and industry stakeholders with the final program governed by ‘getting to know you’ conversations with program dyads, and a steering committee which included four researchers from different disciplines (CM, LdP, JB, GRH), one artist (KM) and one dementia carer (GR).

### Sampling and Recruitment

As this was a qualitative pilot project, sampling was purposive (Palinkas et al., 2015) and convenient. Recruitment for the co-design workshop in *Phase One* occurred through a dementia-friendly group that was led by one of the research team (GR). Consent forms and participant information sheets were distributed to regular attendees of the group by email inviting them to attend and explaining the purpose of the workshop. Eligibility included an interest in community programs for people living with dementia and their family carers. When people arrived at the workshop, the research was explained to the prospective participant and understanding checked prior to signing the consent form.

For *Phase Two*, recruitment for participation in the programs and the research occurred through the distribution of a flyer with the local government, Dementia Australia, some aged care providers, and at the dementia-friendly group where the co-design workshop occurred. To be eligible to participate, community dwelling dyads needed to be a person living with dementia and their family carer. At this stage, some snowball sampling occurred (Palinkas et al., 2015). Potential participants volunteered interest by contacting the lead researchers and they were then sent the participant information sheet and consent form to review in advance of the ‘sign up day’.

### Consent Processes

For both phases of the project, a process of co-consent was followed using a conversational assessment for the person with dementia (Dewing, 2008). This process supported the building of trust and aligned with the collaborative nature of the project. At the sign-up day, the project was explained to each dyad by members of the research team who have experience and skill communicating with people with dementia (CM, LdP, JB, GRH). Also at this time, consent was sought from the family carer and person with dementia. This involved a conversation between one of the researchers and the dyads to explain what they were consenting to, and checking understanding of the research and their ability to communicate their choice to participate, which was evidenced by them saying in their own words what they were agreeing to (Hegde & Ellajosyula, 2016). Once understanding and ability to choose to participate was established, there was the option for the researcher to sign the consent form on behalf of the person with dementia, but this option was not required for any participants. At the conclusion of phase two, consent was also sought from the arts and well-being facilitators who participated in the focus group.

### Data Collection

#### Co-Design

Prior to the co-design workshop the steering committee decided on the rough workshop structure and the questions to ask participants. The workshop began with a presentation giving an overview of the proposed programs and then participants were asked their opinions about arts and well-being activities that would be enjoyable and support them to feel safe during their participation. This process occurred in four groups with a question asked at each table and a researcher sitting at each table (CM, LdP, JB & KM). The groups rotated between tables every 15 minutes. The conversations were recorded using a digital voice recorder on the tables and participants and researchers also wrote notes on large pieces of paper. These conversations were followed by morning tea and general networking. Prior to the programs at the ‘sign up day’ people engaged in a ‘getting to know you’ conversation to understand what their interests were and what they wanted to achieve from program participation. They also participated in a simple art activity and a social morning tea.

#### Perspectives about Concurrent Programs

After every program session, the facilitators completed a reflection which also served as data. At the completion of the programs the dyads participated in a semi-structured interview which was recorded and transcribed verbatim. Due to their shared experience, the interviews were conducted with the dyad either at the community centre or in the dyad’s home. These interviews were completed by an occupational therapist (GRH) with experience working with people with dementia and their family carers and who was not involved in the delivery of the program. Whilst the literature suggests that conducting interviews with dyads can be complex, the interviewer found that for this topic, having the carer present provided support to the person with dementia, and offered prompts (Novek & Wilkinson, 2017). The focus group with art and well-being facilitators of the programs was convened at the community centre by CM and LdP to ask them for feedback about their experience. It was recorded and transcribed verbatim. The interview questions with the dyads and focus group questions for program facilitators are available in table 1.

**Table 1.** Interview and Focus Group Questions

After program completion – interview with participants
(can be completed in dyads with person with dementia and carer if they would like to) 1. In your opinion, how do you think the program went? 2. What do you think were the best things about the program? 3. What did you get out of participating in the program? 4. What facilitated the delivery of the program? 5. What impeded the delivery of the program 6. If you had the opportunity, would you participate in such a program in the future? 7. Was there anything else that you wanted to share that you haven’t had a chance to?
After program completion – focus group with art and wellness facilitators
1. In your opinion, how do you think the program went? 2. What do you think were the best things about the program? 3. What did you observe about the people who participated? 4. What facilitated the delivery of the program? 5. What impeded the delivery of the program 6. If you could deliver the program again, what would you like to do differently next time? 7. Was there anything you would like to share that you haven’t had a chance to yet?

### Data Analysis

#### Phase One

Following the co-design workshop, GRH listened to the workshop recordings and cross-checked these with the contents on the table notes. The contributions from the participants were collated for the steering committee to review and further decisions were made about the structure and content of the group programs. The information from the ‘getting to know you’ conversations and drawings with dyads also informed program content to suit individual needs.

#### Phase Two

Once the programs were under way, the weekly reflections from the facilitators were summarised and collated by GRH for review by the steering committee during the program implementation. Transcripts from the interviews and focus group were analysed using Braun and Clarke’s six stages of reflexive thematic analysis (Braun & Clarke, 2021). The inductive analysis began with researchers familiarising themselves with the data by individually reviewing the transcripts (stage one) and assigning codes and notations to each transcript (stage two). The research team then met to discuss differences across interpretation and to reach agreement using a whiteboard to conceptualise six candidate themes (stage three). Transcripts were then uploaded to NVIVO and further coding occurred by CM to review the six candidate themes (stage four). These were then written up into descriptive summaries with supporting quotes by CM and further reviewed by the research team. After further refinement and reconceptualization three overarching themes that included the perspectives of both facilitators and dyad participants were established (stages five and six). Details of the audit trail showing the thematic analysis iterations can be found in table 2.

**Table 2.** Audit Trail Showing Thematic Analysis Process

Code	Iteration 1	Iteration 2	Iteration 3
“didn’t feel hassled”	Theme 1: Living in the moment, relaxing, friendly	Theme 1: A chance to relax • Living in the moment • Separation and safety	Theme 1 relaxation, engagement and trust • Living in the moment • Separation and safety
“He built that trust up” “The safe environment”	Theme 2: Separation and feeling safe		
“he’s an artist that we didn’t know about”	Theme 3: Developing new attributes	Theme 2: A new outlook	Theme 2: Recognising and developing skills and abilities
“Having good social contact”	Theme 4: Interactions, connections, chatting – shared experience • Company of others Connecting, shared concerns and reminiscence	Theme 3: Connecting over shared experiences • Positivity and having fun • A friendly community	Theme 3: Connecting over shared experiences • Positivity and having fun • A friendly community
“Actually supporting each other”	Theme 5: Everyone in the community, companionship		
“Something to help us”	Theme 6: Interpreting experience through carer stress	Theme 4: Diversity in interpretation of experience	Subsumed into other findings

### Rigour

Procedural rigour in data collection was strengthened through having pre-established interview and focus group guides. Credibility in findings was enhanced through having an impartial person (GRH) who was not a member of the steering committee nor a facilitator of the program summarising the data collected from the co-design workshop and completing the interviews. Credibility was also strengthened by having both dyads and program facilitators participating. The steering committee all participated in analysis thus increasing confirmability in interpretation of findings and supporting quotes are used to illustrate the findings which adds to their dependability (Guba & Lincoln, 1994).

### Authorship Position

The research team all have experience with people living with dementia and carergivers. Four of the team are experienced researchers with PhD qualifications (AB, CM, JB & LdP); the research team consisted of three occupational therapists (AB, CM, GRH); one social worker (LdP); one psychologist (JB); an artist (KM) and a dementia carer (GR). The team all had shared values around the importance of psycho-social programs for supporting people with dementia to live well in the community for longer.

## Findings

### Phase One – Co-design of Pilot Programs

Twenty-one people participated in the co-design workshop, including eight males and 13 females. Participants were one advocate from local council, one advocate from Dementia Australia, three people with dementia, four carers and 12 who did not reveal if they were a caregiver or a person with dementia. Participants gave feedback on the proposed program structures. They questioned the appropriateness of cooking and hypnosis for caregivers and the possibilities of spritzers triggering allergies. There were requests for focus on ‘doing’, rather than talking. In response it was decided that the cooking session would focus on the creativity of the different ingredients rather than the cooking process itself, that the hypnosis session be reframed to ‘relaxation and hypnotherapy’ and that allergies be allowed for in planning. For the art program, there were requests for flexibility based on interest and creativity, whilst also having structure, demonstrations and examples. There was also a request for soft music to be played and opportunity given to connect with nature for both groups. Other feedback related to the group process itself and was organised under four headings: ‘set up for success’; ‘being at ease’, ‘new experiences with others’, and ‘individual needs’. Selected quotes to explain this feedback are provided in table 3. One co-design participant described the programs as being a ‘win-win’ for people with dementia and their family carers:

“if it’s going to be good for the diagnosed person, then it’s got to be fantastic for the carer because that gives them a break from the onerous duties of looking after the diagnosed person in that particular moment, so it’s got to be a plus.” (participant in co-design workshop)

**Table 3.** Selected Quotes From Co-design Workshop

Set up for success	“Find the right time of day to do it” “Like watercolour as it is more forgiving” “…Need to know what’s happening to know what to bring…an old shirt or painting top…” “Start within comfort zone” “Everyone can get away with doing abstract painting” “Don’t want to feel inadequate” “Don’t want art to feel threatening”
Being at ease	“Sometimes it feels like your life becomes a series of medical appointments and family dynamics can become challenging, so want a place where you can come and relax” “Group hypnosis sounds risky” “Use of the word hypnosis is bad for this group” “Have a feeling that your thoughts will be respected” “Keep out religion and politics”
New experiences with others	“Need to have more than just expecting people to talk because they may not be in a talking mood, but I think you do need some new experiences so you don’t get bored” “I want to create something meaningful” “Colouring in or more abstract art could be more fun” “I’m at a point in my life that I am willing to give everything a go” “it’s about quality of life” “Fill a void that is sometimes only by being with other people”
Individual needs	“…Dementia looks so different…” “Mandala could be frustrating, too structured” “Sensory experience” “If there’s a sense of perfection it’s too hard for me” “I’d like to learn some resilience I’m starting to yell” “Some people need to be shown or some initiative in what to do…somebody has to start it”

### Description of Concurrent Programs

Based on the co-design workshop, the intent was for both programs to include ‘doing’ activities rather than being focused on discussion or education (see Table 4 for overview of the programs). The sessions were 120 minutes long including morning tea. The art program for the people with dementia had an opportunity to try five different styles of art with the final week being a choice based on what the group decided. There was one consistent facilitator of the art program and one consistent volunteer. The well-being program had a different topic each week delivered by an expert facilitator. As the topics required different skill sets, there were four facilitators overall who delivered the six sessions for the family carers. There was also another volunteer who moved between the groups as needed and arranged morning tea.

**Table 4.** Overview of the Concurrent Program

Art Program	Well-being Program
Seascape painting in acrylics	Cultural cooking
Landscape painting with watercolours	Self-care with accupressure hand massage and gentle movement
Fun with inks	Resilience
Mandala drawing using template with music	Nuture your hands, gentle movement and meditation
Your choice – choose your inspiration (choice of acrylics on easles)	Relaxation and hypnotherapy for managing stress and anxiety
Connect to nature – combined session with nature walk and seed paper	Connect to nature – combined with nature walk and seed paper

### Phase Two – Qualitative Perspectives

Six spousal dyads participated in the programs making 12 people overall. One of the dyads were living separately in the community but remained in a caring relationship. Five of these dyads were from the dementia friendly group and one dyad heard about the group through other means. There were three males and three females in the art group and three males and three females in the well-being group for the family carers. The age range for family carers was 72 – 81 years and the age range for the people with dementia was 73- 78 years. Reasons for wanting to participate included meeting new people, to relax and for something to do. Six female facilitators participated in the focus group.

General feedback about the program structure and content was that participants enjoyed the resilience session, the acupressure and learning about self-care. There were mixed views about the hypnotherapy and relaxation session with feedback that there was too much talking and not enough doing. The cooking session may have been better received as a later session rather than first and with clearer explanation of the session purpose (i.e., people didn’t need to cook but could simply enjoy the experience). Consistent with what participants said in the co-design workshop, the family carers enjoyed sessions when they were interactive and informal and did not require a lot of listening or written materials. Feedback about the content of the art program from the people with dementia was that they enjoyed the chance for free choice in session five, opting for acrylics on the tabletop easels. The combined activity and nature walk in the last session was also well received and described by facilitators as a positive way to finish.

Three themes were identified that represent both the dyads and facilitator perspectives about engagement and participation in the program. Theme one ‘relaxation, engagement and trust’ includes two sub-themes and describes how participants felt increasingly safe and comfortable about being separated from their partner. Theme two ‘recognising and developing new skills and abilities’ reflects the insights about ability and opportunity that were generated through program involvement. Theme three ‘connecting over shared experiences’ includes two subthemes that explain how participants had fun and made new friends. Quotes attributed to family carers (FC) and people with dementia (PwD) are provided with a pseudonym to protect identity. Pseudonyms align with identified gender of participants (see Table 5 for dyads). Facilitators are not identified with individual quotes but rather indicated as focus group (FG).

**Table 5.** The Dyads, Their Pseudonyms and Gender

Person with dementia (*n* = 6)	Family carer (*n* = 6)
Tom (M)	Pat (F)
Winnie (F)	Michael (M)
Andrew (M)	Phyliss (F)
Cameron (M)	Audrey (F)
Sharon (F)	Greg (M)
Susan (F)	Scott (M)

Abbreviations: F = female; M = male; n = number.

### Theme 1: Relaxation, Engagement and Trust

The group process was a chance for participants to ‘switch off’ and relax. Being able to relax appeared to relate to becoming engaged in the activities and feeling safe in the groups. Feelings of safety were not immediate but grew across the six weeks of the program. Feeling safe whilst being separated from their partner relied on developing trust in the program facilitators. Having this trust enabled participation, connection and sharing with other group members and facilitators.

#### Sub-Theme 1.1 Living in the Moment

After arriving and settling into the groups, the facilitators noted a shift in mood as participants became immersed in ‘doing’. One of the facilitators described this process of engagement and not focusing on the outcome.

“I was talking through with him (PwD) what the options were … getting him to select colours and only helping him if he really couldn't finish a particular task…he was enjoying that process … I didn't want to push it too far because it's about the enjoyment and about his engagement, not about doing the picture.” (FG)

Sharon was appreciative of being able to participate on her terms and in her time.

“nobody hassled … if you go at your own pace, you can do” (Sharon, PwD)

There was a reflection from Tom (PwD) and Pat (FC) that becoming immersed in the art helped to distract from other concerns.

“Pat (FC): Tom (PwD) often gets headaches … one day when we were coming, and he was complaining about headache and I said well see how you’re like afterwards and he said the headache did clear. So, it's taking his mind off the things around him

Tom (PwD): it’s usually there for just a while, right bang in the middle with the headache.

Pat (FC): he found that the arts … helped that day … it helps take, takes his mind off other things and the companionship. You seem … to quite enjoy.

Tom (FC): Yeah, I enjoyed it. It was good.”

In the art group, participants connected through music that was playing while they painted – they would comment on the genre and having choice about what music to listen to. As the facilitators got to know the PwD better, there was opportunity for reminiscence as well.

“I just love seeing our [art] group having so much fun and the connections as well … we had quite a bit of banter … lots of sharing of different songs that they liked…. let's beat it up a little bit and get some tunes going … we really did have fun in there…”. (FG)

#### Sub-Theme 1.2 Separation and Safety

*“Building up the trust”* (FG) so that the structure, expectations, and environment felt familiar meant participants became comfortable separating from their partners and knowing that they were cared for - but nearby. The feelings of safety developed across the six weeks and were described by FG facilitators as being *“comfortable*”, “*knowing familiarity*”, and having “*connection.”*

“from the very first session, there was quite a lot of anxiety there and I did see that really shifted by the end, and everyone was so much more relaxed, you know, probably by the second or third session in.” (FG)

“The connection… And the support. The safe environment, I think they knew it was safe and I think they knew they weren't being judged. That was …key.” (FG)

The feelings of safety manifested for one of the PwD with anticipation of the group, even though he could not remember the details.

“I don't know if it was an ESP [extra sensory perception] because he’s not got time or place anymore and he was saying, can we go, can we go? So, he was obviously, had some sense of anticipation, I think of the class happening.” (Audrey, FC)

Audrey (FC) also gave feedback that the skills of the facilitators in ensuring her partner (Cameron, PwD) was safe and settled during the program helped her to feel comfortable; *“they needed to keep a watchful eye”* and she appreciated that the facilitators focused on *“what people do best rather than try and do a generic thing that didn't hit the mark”*. Knowing their partner was getting something out of the group, meant FCs could focus on their well-being during their program and recognize their needs.

“I think they recognized how much [they needed] … a break and how much limited support they have … the demand is on them to be the supportive one” (FG)

“I know for some … they really enjoyed having that bit of separation where they could do something for themselves because they don't get that much in their day-to-day life”. (Pat, FC)

The safety led to the FCs being *“a group of people who were very prepared to try all sorts of things they hadn’t tried before”* (FG). They were “vulnerable in their sharing” (FG) and provided examples of *“what they've done that's worked for them, and they were very open with that”* (FG). In particular, the facilitators agreed that the resilience session was *“really beautiful”* (FG).

“we talked a lot about emotions in my session, so grief came up … I think just being told that … those emotions are there for a reason … felt really heard and … they couldn't stop the conversation …”. (FG)

Similarly, for the art group, the facilitators acknowledged how people can feel vulnerable around art but that *“they did seem to feel safe”* and *“they don’t worry about what we’re asking them to do”*. The facilitators also noted that when the dyads rejoined after their sessions, there was a positive mood as they reconnected with each other after their separate experiences.

“coming in and showing what they've done in their artwork at the end - the mood changed. It was, it was genuinely everyone was looking at other peoples and giving affirmations and commenting” (FG)

### Theme 2: Recognising and Developing Skills and Abilities

This theme has two aspects. Firstly, FCs shared their observations and new learnings about the abilities of their partner (PwD) to engage in the group and participate in the art. Viewing the PwD enjoying themselves gave the FCs and facilitators pleasure and they began to view them in a new light.

“could see a lot of things coming out and she was quite happy, she enjoyed it which is the best part for me that she was enjoying … so I got enjoyment from that side” (Scott, FC)

The FCs expressed some surprise at what their partners and the whole group were able to achieve through their participation in the art program, especially as many of them had not done too much painting before.

“Andrew really enjoyed the painting, he’s an artist that we didn’t know about” (Phyllis, FC)

“he said he had absolutely no idea how much of an artist his wife was … he's like, I had no idea … so even the carers learnt something about their partners too” (FG)

“From a carers perspective, I think it's very successful … with the end results, and some of the things that came out of it were quite astounding, and quite bloody beautiful some of them, some of the paintings were magnificent” (Michael, FC)

Michael (FC) further described his wife as being *“a bit hesitant at doing it”* but then *“when her imagination got to work on it”* what she created was *“unbelievable”*. He was pleased that she was being stimulated in this enjoyable way for her. There was pride in the artwork that participants made through the program and which they *“put up on the wall”* (Audrey, FC). When Sharon (PwD) saw her artwork, she laughed with happiness and said, *“it was so lovely … give it [artwork] to me.”*

“One of the really good things I think about the program was that several of them actually took pieces home and kept working on them and brought them back to shows us … there was a few that were quite happy with having the nice comments from their family” (FG)

Secondly, participants described how learning from the programs translated into day-to-day life for dyads giving them a new outlook. Facilitators noticed FCs learning more about themselves even when they didn’t expect to.

“a couple of people [carers] who didn't think they were going to learn anything new … were quite surprised, that there were things that they didn't consider or aspects they hadn't heard before”. (FG)

This translation of the art program to changes at home for the PwD was described by Susan (PwD) who found the art has helped her learn to “*relax”* more.

“… I actually go home now and I … do a bit of colouring in … I've done some things on pots and yeah with acrylic paint and so I have got something out of it because I never used to do that. I wouldn't sit … I can actually sit and I try and read a bit too. Yeah, so it's helped me, I think, to relax a bit more”. (Susan, PwD)

Phyllis (FC) described the program as enabling both her and her partner to *“extend our horizons”* and it gave them *“something to look forward to”*. The carers also continued with things they learned in their self-care program at home.

“I think just the fact that they went and continued on with what we had taught them and yeah, you know, doing acupressure and all that sort of thing” (FG)

“I try to use the acupressure … I wake up at night … and I can't get back to sleep and find if I do some acupuncture (sic) or try and like … just relax … so that's helped”. (Phyllis, FC)

Once the program finished, the realisation of the program’s value led the FCs to look for something else that would satisfy this creative need for their partner.

### Theme 3: Connecting over Shared Experiences

This theme describes the community culture that was established in the groups where there was positivity, and friendships formed through the support they gave each other and that they received from the facilitators. Some of the participants already knew each other; however, they got to know each other better through the program and made new friends as well.

#### Subtheme 3.1 Positivity and Having Fun

There were connections established each week through shared *“commonalities”* (FG), hearing other *“points of view”* (Greg, FC) and providing support. The ‘doing’ aspect of the program meant the focus was on having fun with the activities, with their problems being *“sort of peripheral to that”* (Greg, FC).

“it's just different … I really enjoyed it and meeting the new people in the same situation… so I'm grateful… And it was just fun… it was so interesting”. (Phyllis, FC)

“being with each of the carers and just seeing each of the conversations that they were having and their interactions, but also the support that the group brought to each other, or that the program brought” (FG)

The facilitators suggested that the dyads may have been lonely and isolated meaning they established “*that common bond really quickly”* (FG).

“because it is specifically for carers and for people with memory loss … they're all each needing that support because a lot of them are quite isolated, have lost their friends or family and supports and things”. (FG)

Those in the art group demonstrated shared pride in holding up their artwork, helping each other, having photos together and giving encouragement to each other.

“the [art] participants actually supporting each other throughout as well, and encouragement, lots of … really positive comments on each other's work and … they were all looking out for each other.” (FG)

#### Subtheme 3.2: A Friendly Community

In the focus group, the facilitators described the supportive culture as a *“tone”* they were trying to foster so participants could focus on building friendships and successes rather than *“creating the world’s greatest masterpiece”* (FG). The facilitators incorporated flexibility and were responsive to requests from the participants.

“we had some flexibility, which was fantastic, we (well-being group) ended up out under the trees … you know, life happens, we were flexible and we worked with it” (FG)

“making sure they were comfortable and trying to make it fun for them was a really good strategy … there were a couple of things where people stood out or were, you know, didn’t participate in every aspect … but largely they were very receptive and did give everything a good go … the supportiveness across the two groups was quite extraordinary.” (FG).

Scott (FC) reflected on the mix of people and wondered how the program would have been with a different group dynamic. Audrey (FC) agreed that the experience was *“lovely being with this group of mates”*- describing it as *“consolidating … in terms of friendships.”*

“We enjoy it. We like the company. We like the people … I've made a couple of new friends … one of them is a bit mischievous and gets me into trouble.” (Andrew, PwD)

Tom (PwD): “Just the interaction with people here … everybody just to see and whatever you know (sic)”.

Pat (FC): “Yes, I’d agree interaction with other people in the same situation and- “

Tom (PwD): “People who are watching together”

Greg (FC): “getting together with all the people … and having a good social contact”

Sharon (PwD): “yeah that was good”

One of the PwD (Susan) explained how she found the group *“easy*” which is not normally her experience (and her partner Scott had noticed the same).

Susan (PwD): “I'm very hard to mix with people and I found it … was really easy to…”

Scott (FC): “She’s shy and never mixes, but … she's different here with all these people. She talks …”

“he (Scott, FC) said that she's (Susan, PwD) never, this is the first time in her whole life that she's made friendships and he was just blown away by the program… he said never in her life did she have friendship, so this is the first.” (FG)

The facilitators attributed the program’s success to the co-design development and the tailored program structure and could see it addressing the need for social connection and decreasing loneliness in this population.

“I reckon it was just life changing for them. You know, to get that tension support and feel valued”. (FG)

## Discussion

Based on the findings from the research, the *phase one* aim to co-design programs that promoted social support and provided a creative outlet for participants was achieved. The aim for *phase two* to explore perspectives about engagement and participation in the concurrent programs was represented through three themes. Theme one was about engagement within both groups which was facilitated through having trust and being able to relax. Theme two was about how participation in the activities (particularly for the PwD) showcased creative abilities which for some was unexpected and had a flow on to the home and other settings. The community described in theme three was a pivotal ingredient to engagement and participation. The culture created by the facilitators within the groups was one of positivity, fun and social support.

The co-designed element of the project ensured the people for whom the programs were intended influenced the content - meaning the programs were delivered based on their preferences. The value of co-design for foregrounding the voices of people living with dementia and their caregivers is becoming increasingly recognised as necessary and beneficial (Lord et al., 2021; Wang et al., 2019). A key aspect arising from the co-design workshop was the need to set the programs up for success through tailoring the program to individual needs and putting people at ease. There was a desire for engagement, but also an element of worry in the workshop that the programs could be “*risky*”, or they may feel “*inadequate*” (see table 2).

The Alzheimer’s Society (2022) in the United Kingdom advocates for having environments that are perceived as safe and supportive as people with dementia may have lost confidence and can worry about failure, leading to withdrawal. A key component of success appears to be the skills and knowledge of the program facilitators who can offer activities that are graded in complexity to suit individual needs and abilities (Humphrey et al., 2017). Similarly, whilst participants appreciated the paintings they produced, the focus facilitators placed on the process of doing rather than on the outcomes was pivotal for supporting feelings of safety and fun (Cavalcanti Barroso et al., 2022; Letrondo et al., 2023). Letrondo et al. (2023) advocated for art programs being delivered by artists with experience and training working with people with dementia. For the concurrent programs in this project, the small group sizes, the venue, expertise, training, and communication skills of the facilitators were all key to this success; all of which are supported in the literature (Humphrey et al., 2017; Killick, 2012).

The trust described in theme one stemmed from both the skills of the facilitators, and the concurrent design of the programs. Having family carers nearby and involved has been identified as pivotal for putting people with dementia at ease so they can engage with the painting (Irons et al., 2020). Family carers also benefitted from the concurrent design as they could access the program without guilt and fear of separation (Giebel et al., 2023; Leocadie et al., 2018). Family carers described being able to relax and express their grief and stress without judgement whilst knowing the person they care for was concurrently having an enriching and authentic experience. Participation was also likely enhanced due to there being no cost of the programs (Leocadie et al., 2018).

The benefit for the dyads from the programs was realised through recognising and developing new skills and abilities (see theme 2). For people living with dementia, creative processes have been described as evoking memories, improving communication fluency, improving mood and self-esteem, reinforcing identity, and strengthening relationships with partners and arts facilitators (Letrondo et al., 2023; Schneider, 2018). In this case, there was discovery of new talents for the person with dementia and family carers experienced pleasure from seeing their partner engaged and participating in art activities. Multi-faceted approaches have been shown as effective for reducing caregiver stress (Jackson & Browne, 2017). In this case, family carers learned new ways to care for themselves, and to look for creative outlets beyond the programs. It is likely that this level of participation would not have been possible without the co-design element upfront and the trust in the facilitators (as described in theme one).

The social support that the programs offered was possibly due to the culture within the groups which provided informal opportunities for sharing, whilst having new experiences and fun (as described in theme two). Comparatively, research exploring the benefits of ‘Memory Cafes’ also found the value in social connections, but as dyads attended the same group together and they were discussion based (rather than doing), there were some concerns about the possibility of those living with dementia being “ignored socially” whilst their caregivers had their support needs met (Protoolis et al., 2022, p. 365). This finding lends support to the model of having concurrent opportunities for the person living with dementia to connect with others and have their own experience while their family carer received respite, support, and self-care nearby.

A limitation of the pilot was the homogeneity of the dyads who participated with them all being of similar age, and from the same metropolitan city within South Australia. Details of socio-economic status, and ethnicity for the dyads was not collected. Details of the stage of dementia were not collected as this did not seem to align with the focus of the project on abilities rather than disabilities. It is recommended that future programs and research include participants from different locations (i.e., rural) and with recorded diversity of cultural background, and socio-economic status. Similarly, the age, socioeconomic status and cultural backgrounds of the program facilitators was not collected. The facilitators were all female, and it is possible that participants may relate differently to male facilitators. Conducting the interviews with dyads rather than with individuals may have assisted the person with dementia to participate through having their family carer present; although there is also the possibility this process may have limited openness to speak freely (Novek & Wilkinson, 2017).

The main strength of this pilot was that the programs were co-designed with people with dementia and their family carers in *phase one*, some of whom were willing to trial the concurrent arts and well-being programs and provide feedback. Continuation of the co-design and action research approach into *phase two* would have strengthened the pilot and this is a recommendation for further program implementation and research. The researchers involved had experience in working with people with dementia and were able to gain trust with dyads through both phases by seeking opinions, preferences, and feedback. The researchers and steering committee came from diverse backgrounds. They worked as a team and reflected on the process and data to ensure that findings were trustworthy and reflected the experience of participants. Similarly, the arts facilitators were experienced in adapting art-based processes to the abilities of diverse participants, while the family carer and well-being facilitators were open to exploring carers’ needs, providing a safe space to discuss stress and grief as well as trying different activities. One author (GR) is a dementia carer and provided lived experience insights and consumer perspectives throughout the research process.

Arising from this research are some recommendations for community programs for people living with dementia and their caregivers:

(1) More options are needed for accessing respite with the following characteristics:
  • Pays attention to the needs of both the caregiver and the person with dementia.
  • Is designed in partnership with participants and facilitators.
  • To encourage safety and fun, provide opportunity for ‘doing’ activities where there is a focus on the process of participation rather than the end point or product (Humphrey et al., 2017)
  • Small groups that allow time for social connection
(2) Programs are delivered by facilitators with training in communication skills for people with dementia, are skilled in person-centered approaches and are sensitive to the different needs.
(3) Ensures a safe and trusting environment through designing programs that keep caregivers and the person living with dementia close by but provide both groups with an experience tailored to their needs.

Offering programs in this way may increase uptake of respite services. Models of respite and the outcomes of respite have been identified as a priority for future research (Cognitive Decline Partnership Centre, 2016). If programs are attractive, accessible and affordable, then caregivers may seek support earlier thus supporting their well-being. The concurrent programs provide an innovative example of how respite can be reinvented and how art and well-being can be used as a means for dyads to access social support, whilst engaging in stimulating activities, learning more about themselves and having their different needs met.

## Conclusion

Given the prevalence of people living with dementia in the community with caregivers, there is scope for innovative programs that provide social support and well being for dyads. These co-designed pilot concurrent art and well-being programs demonstrate that dyads enjoy having opportunities for connecting with others, sharing experiences, and learning more about their abilities and interests. The respite model of having the art program for people with dementia running alongside and co-located with the well-being program for their family carers showed that dyads readily relax and trust knowing they are engaged and closeby. Concurrent art and well-being programs for people with dementia and their caregivers must have small group sizes that allow for social connection. Trained facilitators provide opportunities for participation in art and well-being activities without focusing heavily on outcomes.
